# Dr. M. McCarty

**Published:** 1875-06

**Authors:** S. J. Cobb

**Affiliations:** Chairman Committee on Memorials, Tennessee Dental Association.


### ARTICLE III.

### *Obituary.*

*Dr. M. McCarty*, of Pulaski, Tennessee, was born October 26, 1832, in Clark ocunty, Kentucky, and was educated at Bethel College.

In the year 1864, he moved from Russellville, where he then resided, to Toronto, Canada, where he studied dentistry with Dr. Elliott. He returned to Russellville in the spring of 1865, and continued the study of the profession with Dr. Jones, of that city.

On the 26th of October, 1865, he was married to Miss A. P. Stewart, and afterwards, during the years 1866 and 1867, he attended one course of lectures at the Cincinnati Dental College. In May, 1867, Dr. McCarty moved to Pulaski, Tenn., where he practiced his profession until January 1, 1874, when he returned to Russellville, where he died in the March following.

Dr. McCarty was a member of the Tennesse Dental Association. He was a quiet, unassuming gentleman, honest and thorough in all his professional work, and but for his short life and feeble health, he would have attained a high order of excellence in his profession.

S. J. COBB,
*Chairman Committee on Memorials,*
*Tennessee Dental Association.*

